# Alcohol consumption and health-related quality of life in regional, rural and metropolitan Australia: analysis of cross-sectional data from the Community Health and Rural/Regional Medicine (CHARM) study

**DOI:** 10.1007/s11136-023-03522-x

**Published:** 2023-10-25

**Authors:** Lisa Redwood, Karli Saarinen, Rowena Ivers, David Garne, Paul de Souza, Andrew Bonney, Joel Rhee, Judy Mullan, Susan J. Thomas

**Affiliations:** 1 Graduate School of Medicine, Faculty of Science, Medicine and Health, University of Wollongong, Wollongong, NSW Australia; 2 MIND the GaP, University of Wollongong, Wollongong, NSW Australia; 3 Nepean Clinical School, University of Sydney, Kingswood, NSW Australia; 4 Discipline of General Practice, School of Population Health, UNSW Sydney, Kensington, NSW Australia

**Keywords:** Alcohol, Quality of life, Gender, Rurality, Australia

## Abstract

**Background:**

Relationships between alcohol consumption and health are complex and vary between countries, regions, and genders. Previous research in Australia has focused on estimating the effect of alcohol consumption on mortality. However, little is known about the relationships between alcohol consumption and health-related quality of life (QoL) in Australia. This study aimed to investigate the levels of alcohol intake and QoL in males and females in rural, regional and metropolitan areas of Australia.

**Method:**

Participants (*n* = 1717 Australian adults) completed an online cross-sectional study. Males and females were compared on measures including the AUDIT-C and WHOQOL-BREF. Data were stratified into risk of alcohol use disorder (AUD) and associations were examined between alcohol consumption and QoL, adjusting for sociodemographic variables.

**Results:**

Males had higher alcohol consumption and were at greater risk of AUD than females (20% vs 8%). Relationships between alcohol consumption and QoL were positive or non-significant for low–moderate AUD risk categories and negative in the severe AUD risk category. Males in regional communities reported higher alcohol consumption (AUDIT-C score 6.6 vs 4.1, *p* < 0.01) than metropolitan areas. Regression analyses identified that after adjusting for sociodemographic variables, alcohol consumption was positively related to overall, environmental, and physical QoL and general health.

**Conclusion:**

The results indicate that alcohol consumption is negatively related to QoL only in those with severe risk of AUD. Males in regional areas reported higher alcohol consumption than those in metropolitan areas. These results provide further information about relationships between alcohol intake and health in Australia that can help inform prevention, screening and delivery of interventions.

## Introduction

Alcohol use is a major risk factor for the global burden of morbidity and mortality [1]. Hazardous alcohol intake can cause short-term harms associated with acute intoxication and long-term harms associated with chronic overuse, which represents 5.3% of deaths globally [2]. Despite this, relationships between alcohol use and health are complex and multifaceted, with moderate alcohol consumption having potentially protective effects on some conditions [1]. Many studies find J or inverted U-shaped relationships between alcohol and health outcomes, with moderate drinkers having higher QoL than heavy drinkers or abstainers [3].

Quality of Life (QoL) is an important indicator of overall wellbeing. Frequent and excessive alcohol intake may impact an individual’s QoL [4–7]. Due to the complex relationships between health and alcohol, a useful approach is to consider alcohol intake in relation to multifaceted measures of QoL which assess subjective health and wellbeing in distinct domains such as physical, psychological and social functioning. This approach allows for a more nuanced understanding of negative, neutral, and potentially positive aspects of alcohol consumption at different levels in relation to different domains of functioning.

Several international cross-sectional studies found significant differences in QoL between alcohol-dependent participants and non-alcohol drinkers [4, 8, 9]. Trifkovič et al. found that Slovenian participants with alcohol dependence were less satisfied with their QoL when compared with non-alcohol-dependent participants [5]. Two cross-sectional studies found that harmful or hazardous consumption, including binge drinking, were related with lower QoL among young adults in Europe [7, 10]. Previous research has demonstrated that there may be no difference in overall QoL between alcohol-dependent and non-dependent participants while at the same time, there are significant differences for some specific QoL domains [5]. The results from these findings cannot necessarily be generalised to Australia due to global socio-economic and cultural differences in alcohol consumption [7].

Relationships between alcohol consumption and QoL in Australia have received very little attention. An Australian study conducted in 2018 reported distinct variability in QoL with most people living with alcohol use disorders reporting low-to-moderate QoL [11]. It also found that recent alcohol consumption (within the 30 days prior to the assessment) was correlated with lower QoL [11]. This study was conducted in people attending treatment for alcohol or other substance use facilities in New South Wales (NSW) and therefore does not reflect the general population [11]. However, a study conducted in rural NSW also reported that people with very-high-risk alcohol consumption have significantly lower QOL than people who consumed alcohol at a low risk level [12]. This study included only NSW residents and therefore may not be generalisable across Australia more broadly. Furthermore, the nuances between rurality and gender were not explored. Additionally, no studies were found that examined QoL and alcohol consumption in the general Australian population. Therefore, more research in this area is required as QoL is an important indicator of multifaceted health and wellbeing, and it is often used as an outcome measure to evaluate the success of alcohol use interventions [5, 13, 14].

There is considerable geographical variation in alcohol use and alcohol-related harm, both between and within countries [1]. The variations are driven by a multifaceted combination of region-specific sociodemographic and economic factors, including the social determinants of alcohol use such as local drinking culture, trauma and mental health disorders [1, 15]. The epidemiology of alcohol consumption is an important public health priority to inform the development and implementation of tailored health strategies.

Alcohol plays a prominent role in Australian culture, and a significant number of Australians exceed the national recommended drinking guidelines [16]. While the number of people reducing their alcohol consumption in Australian is increasing (from 28% in 2016 to 31% in 2019), the level of hazardous alcohol consumption persists, with approximately 25% of people drinking at a dangerous level on a single occasion, that is binge drinking, at least monthly [17]. This is likely an underrepresentation as recent studies have reported inaccuracies in the measurement of alcohol consumption in Australia [18]. Such hazardous alcohol intake places a substantial burden on the healthcare systems due to the increased risk of injuries and chronic medical conditions [19, 20].

Relationships between alcohol use and QoL may differ by gender [21]. Some studies have found stronger negative associations between binge drinking and QoL in males than females [7]. In contrast, Trifkovič et al. found a stronger correlation between women who were alcohol-dependent and lower QoL scores than males who were alcohol-dependent [5]. There is a need for more research into gender differences in the Australian context, as this may assist health care professionals to better understand and reduce harm.

There are considerable differences in alcohol consumption between people residing in rural and urban areas. Hazardous and harmful alcohol consumption increased in rural areas compared to urban areas between 1990 and 2019 globally, with Australia having one of the highest increases [22]. Potential explanations for the increased alcohol consumption in rural areas include perceived social benefits, feeling included and participation in community events [22]. Rural communities also have more severe alcohol-related harms than urban populations, such as increased suicide rates, hospitalisations, cirrhosis, drink driving and road injuries [22–25]. This could partially be explained by lower access to alcohol treatment options and fewer health professionals in rural than urban areas [25]. The current literature regarding alcohol use in rural Australia has mainly focused on the epidemiology and harms of alcohol use. Alcohol consumption and QoL differences between regional and metropolitan Australians have yet to be explored.

To address these gaps, the study aims were to (i) investigate the alcohol consumption and quality of life by gender and alcohol risk category, along with interactions between alcohol risk category and gender on QoL; (ii) quantify the risk of alcohol use disorder by gender; (iii) assess the relationships between alcohol intake and multifaceted QoL in Australia, stratified by gender and alcohol risk categories (low, medium, high, severe); (iv) analyse the differences in alcohol consumption and QoL between respondents residing in major cities, inner regional and outer regional and remote areas; and (v) evaluate the relationships between alcohol intake and domain-specific QoL, adjusting for sociodemographic characteristics.

## Methods

### Study design and recruitment

Data were collected as part of the CHARM (Community Health and Rural/Regional Medicine) project between August 2020 and March 2022, which overlapped with the COVID-19 pandemic. For context, a brief timeline of the COVID-19 pandemic in Australia is that the first case in Australia was recorded in January 2020, with the first COVID-19 wave occurring between March and May 2020, the second between June and November 2020, a Delta wave occurring between July and December 2021 and an Omicron wave during 2022. All states experienced a range of measures to contain the virus including social distancing and lockdowns [26]. This is a cross-sectional, online, health survey aiming to gain a better understanding of the health needs, community issues and perspectives of Australians residing in rural, regional and metropolitan areas. The survey took approximately 15–20 min to complete and covered multiple aspects of health, wellbeing and satisfaction with aspects of life and community. The survey was promoted through social media groups located Australia wide, including those in regional, rural and metropolitan areas. A prize draw of three $100 store vouchers was offered to encourage participation.

### Demographic information

Demographic information, such as age, gender and postcode, was collected. Postcodes were coded into remoteness areas (RA) based on the Australian Bureau of Statistics ‘Australian Statistical Geography Standard (ASGS): Volume 5—Remoteness Structure, July 2016’ [27]. There are five classes of RA that allow consistent analysis over time. These include RA1: major cities of Australia, RA2: inner regional Australia, RA3: outer regional Australia, RA4: remote Australia and RA5: very remote Australia [27].

### Measures

Data were collected using an online self-administered survey that comprised of several validated questionnaires. For the purpose of the current study, the World Health Organization Quality of Life-brief version (WHOQOL-BREF) and the Alcohol Use Disorders Identification Test-Concise (AUDIT-C) [28, 29] were analysed.

### WHOQOL-BREF

The WHOQOL-BREF is a brief version of the WHOQOL-100 [30]. The WHOQOL-BREF contains 26 questions and is used to assess respondents’ health-related QoL across physical, psychological, environmental, and social wellbeing domains [28]. Overall QoL is measured by a single item (Question 1*; How would you rate your quality of life?*). Overall satisfaction with health is measured by a single item (Question 2; *How satisfied are you with your health?*). Each question is scored on a Likert scale from 1 to 5. Domain scores are scaled in a positive direction (i.e. higher scores indicate higher QoL) [28]. The Physical Health domain includes questions about activities of daily living, dependence on medicinal substances and medical aids, energy and fatigue, mobility, pain, sleep and work capacity. The Psychological domain includes bodily image and appearance, positive and negative feelings, self-esteem, spirituality and cognition. The Social Relationships domain covers satisfaction with personal relationships, social support and sex life. The Environmental domain includes financial resources, freedom, physical safety and security, accessibility and quality of health and social care, home environment, opportunities for acquiring new information and skills, opportunities for recreation, physical environment and transport. Participants with more than 20% of responses missing were excluded from analysis [30].

### AUDIT-C

AUDIT-C is a shortened version of the original AUDIT. It was developed by the WHO as a screening tool to help identify individuals that are drinking excessively and are at an increased risk of having an alcohol use disorder [29].The AUDIT-C consists of three questions which measure alcohol consumption over the previous year, with scores ranging from 0 to 12 [29]. Score of zero reflect no alcohol use and higher scores reflect higher alcohol use. Risk categories were then calculated by gender. For males a score of 0–3 was categorised as low risk, 4–5 moderate risk, 6–7 high risk and 8–12 severe risk. For females, a score of 0–2 was categorised low risk, 3–5 moderate risk, 6–7 high risk and 8–12 severe risk [31]. Participants who did not complete all required questions were excluded from analyses.

### Data analysis

Data were collected on Qualtrics™ and statistical analyses were conducted using the Statistical Package for Social Sciences (IBM: SPSS Version 25) and R studio [32–34]. Prior to analysis, data were cleaned by removing participants with fewer than 80% of responses completed. The total number of survey responses was 2416, which was reduced to 1717 after removal of incomplete responses. We employed recommended steps to reduce and identify potential data integrity issues such as computer-generated (bot) responses [35]. Instead of a guaranteed payment, survey completers could enter a prize draw and were required to follow a link to another Qualtrics survey to enter their personal details; this approach has been found to be the most effective way of deterring bot activity [35]. Summary statistics and descriptive analyses were performed to check whether expected values, distributions and relationships were present, while checking for anomalies such as inconsistent responding and exact duplication of responses to free text answers [35]. Additionally, survey respondents were required to enter an Australian postcode, and these were verified by mapping responses to the Australian Bureau of Statistics database. Qualtrics also collects meta-data which provide the general location of the respondent, accurate to the nearest city. Next, we conducted Mahalanobis distance analyses on study variables including time to complete the survey, which is one of the most effective ways of detecting outlier respondents, non-human response patterns and atypical response sets that may indicate careless responding [36].Mahalanobis distance analyses indicated the presence of 14 multivariate outliers in the dataset. The analyses were run with and without the outliers and because the results were equivalent it was decided to retain them in the final analyses.

Two-way ANOVAs were performed to compare AUDIT-C and WHOQOL-BREF scores by gender and alcohol risk category. A Chi-square test of independence was performed to examine the relation between gender and alcohol use risk category. Bonferroni-corrected post hoc analyses were then performed to identify any significant differences in alcohol use risk category between genders [37, 38]. Spearman’s correlations were performed to assess the relationships between alcohol consumption, gender and QoL in Australia. As previous studies have identified that the direction of relationships between alcohol consumption and health may differ for people in different risk categories, correlations between AUDIT-C scores and QoL domain scores were performed separately for participants in each AUDIT risk category (low, medium, high, severe). An ANOVA was used to compare alcohol consumption and QoL by rurality. Lastly, a multiple linear regression was used to evaluate the relationships between alcohol intake and domain-specific QoL, adjusting for sociodemographic characteristics.

Due to the small cell sizes in the more remote areas of Australia, the RA areas were combined to create three categories. ‘Major cities’ included RA1, ‘inner regional’ included RA2 and ‘outer regional and remote’ included RA3—outer regional, RA4—remote and RA5—very remote.

### Ethics

This study was approved by the University Human Research Ethics Committee [ref 2020/271]**.** Participants were provided with full written information about the study prior to participation. Data were collected in a de-identified format.

## Results

### Sociodemographic characteristics

The age range of male participants (*n* = 289) was 18–80 years, with a mean of 51.8 years (SD = 15.2). Female participants (*n* = 1410) accounted for 83% of the study sample, with an age range of 18 to 89 years and a mean age of 50.6 years (SD = 14.1). Across the five RA in Australia, 453 (21.6%) study participants were living in major cities' areas (RA1), 1004 (47.8%) participants were living in inner regional areas (RA2), 260 (12.4%) were residing in outer regional areas (RA3) and 2 (0.1%) were residing in remote and very remote areas (RA4 and RA5).

### Alcohol consumption and quality of life by gender and alcohol risk category

**Fig. 1:**
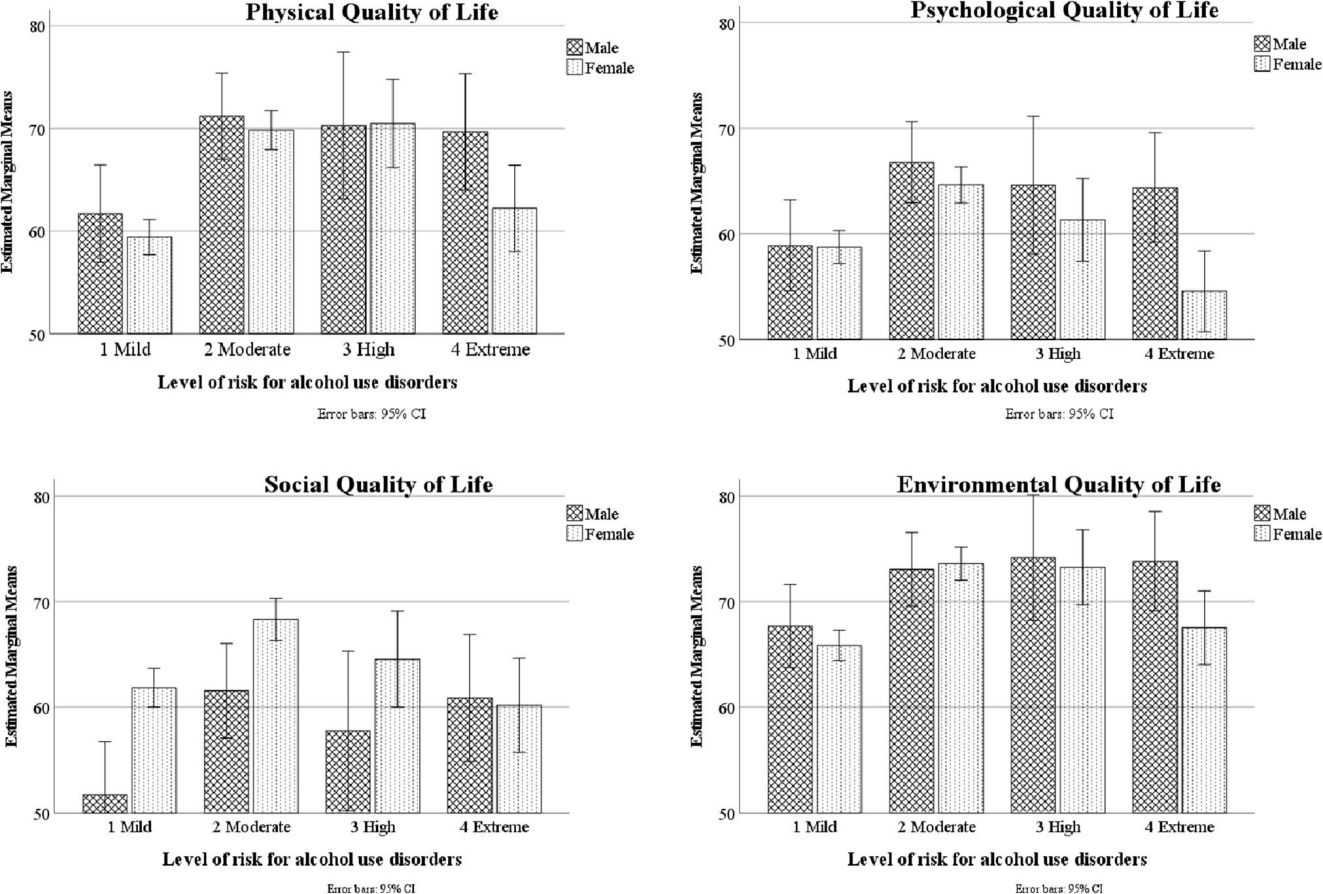
Health-related quality of life domain scores by risk of alcohol use disorders in Australian males and females. Note: Quality of life was measured using the World Health Organization quality of life-brief version, risk of alcohol use disorders was measuring using the alcohol use disorders identification test-concise

Two-way ANOVAs indicated that males had higher AUDIT-C scores than females. There was no significant difference between males and females for overall QoL. Males scored higher than females in general health, physical and psychological QoL. Females scored higher than males for social relationships QoL (Table 1). There were significant main effects of AUD risk category on Audit-C score, overall QoL, general health and all QoL domains (Table 1 and Fig. 1). There were no significant interaction effects between gender and AUD risk category with regard to the study variables.

**Table 1:** Levels of alcohol consumption and QoL by gender and alcohol risk category

	Gender					AUD risk category			Gender AUD risk category*		
	Male M (SD)	Female M (SD)	*F*	*p*	ηp 2	*F*	*p*	ηp 2	*F*	*p*	ηp 2
AUDIT-C score	4.4 (3.1)	3.0 (2.6)	61.98	< 0.001	0.037	3829.42	< 0.001	0.877	1.43	0.23	0.003
QoL											
Overall QoL	4.1 (0.9)	4.2 (0.9)	2.56	0.11	0.002	10.48	< 0.001	0.019	0.97	0.41	0.002
General health	3.5 (1.1)	3.3 (1.1)	4.38	0.04	0.003	7.81	< 0.001	0.014	0.43	0.73	0.001
Domains											
Physical	67.9 (20.8)	64.6 (22.6)	2.76	0.10	0.002	12.17	< 0.001	0.022	0.84	0.47	0.002
Psychological	63.7 (21.0)	61.1 (19.9)	6.59	0.01	0.004	7.33	< 0.001	0.013	1.99	0.11	0.004
Social	57.9 (25.2)	64.3 (23.0)	10.87	< 0.001	0.007	6.53	< 0.001	0.012	1.79	0.15	0.003
Environmental	71.7 (17.8)	69.5 (18.6)	2.43	0.12	0.002	7.89	< 0.001	0.014	1.23	0.30	0.002

*SD* standard deviation, *AUDIT-C* alcohol use disorders identification test-concise, *AUD* alcohol use disorder (1 = Low risk, 2 = Moderate risk, 3 = High risk and 4 = Severe risk), *QoL* quality of life, measured using the World Health Organization quality of life-brief tool (WHOQOL-BREF), *Social* social relationships

QoL domain scores by gender, stratified by alcohol risk category, are plotted in Fig. 1. Across each domain of QoL, generally higher levels of QoL were reported by those with moderate than very high or low drinking risk categories. Bonferroni-corrected post hoc comparisons indicated that Physical Health QoL was lower in the low AUD category than all other categories, and higher in the moderate than the severe AUD category. Psychological QoL was lower in the low than the moderate AUD category and higher in the moderate than the severe category. Psychological QoL was lower in the low than the moderate AUD category, and higher in the moderate than the severe AUD category. Environmental QoL was higher in the moderate and severe categories than the low AUD category. Overall QoL and general health were higher for respondents in the moderate and high than the low AUD categories. General health was lower for those in the severe than the moderate-risk AUD category.

### Risk of alcohol use disorders between males and females in Australia

The Chi-square test of independence showed a strong relationship between the risk of alcohol use disorders and gender, *X*^2^ (3, *n* = 1616) = 860.24, *p* =  < 0.01 (Table 2). The Bonferroni-corrected post hoc analysis identified that males were more likely to have a high (*X*^2^(1, *n* = 1616) = 8.82, *p* =  < 0.01) or severe risk (*X*^2^(1, *n* = 1616) = 41.22, *p* =  < 0.01) for alcohol use disorders than females.

**Table 2:** Comparing risk of alcohol use disorders between males and females in Australia

Gender	Alcohol use risk category				*p* value
	Low* *n* (%)	Moderate *n* (%)	High* *n* (%)	Severe* *n* (%)	
Male	82 (29.4)	104 (37.3)	36 (12.9)	57 (20.4)	< .01
Female	617 (46.1)	516 (38.6)	100 (7.5)	104 (7.8)	

Chi-square

*Significant post-hoc Bonferroni adjusted *p*-values

### Correlations between alcohol intake and domain-specific quality of life

Due to gender differences displayed in Table 2, the correlations between alcohol use disorder risk categories and QoL were performed separately for males and females (Table 3). Overall, the correlations between alcohol consumption and QoL were positive or non-significant in those at low risk of an alcohol use disorder, non-significant or positive for those at medium or high risk of an alcohol use disorder, and negative for those at severe risk of alcohol use disorder, particularly for females.

**Table 3:** Correlations between alcohol intake and domain-specific quality of life, stratified by gender and alcohol risk category

Gender	Risk of alcohol use disorders^~^	Spearman’s correlations between alcohol intake (AUDIT-C Score) and					
		Quality of life (WHOQOL-BREF), stratified by gender and risk of alcohol use disorder					
		Overall QoL	General health	Physical	Psychological	Social	Environment
Male	Low	0.15	0.11	0.08	0.33^a^	0.09	0.08
	Medium	− 0.01	− 0.08	− 0.05	− 0.13	− 0.12	− 0.07
	High	0.11	0.09	− 0.14	0.12	− 0.08	− 0.01
	Severe	− 0.18	− 0.29^b^	− 0.09	− 0.25	− 0.33^b^	− 0.07
Female	Low	0.18^a^	0.09^b^	0.13^a^	0.08^b^	0.08	0.12^a^
	Medium	0.10^b^	− 0.01	0.01	− 0.04	− 0.07	0.05
	High	− 0.06	− 0.14	− 0.07	− 0.03	− 0.02	− 0.08
	Severe	− 0.16	− 0.25^a^	− 0.22^b^	− 0.25^b^	− 0.20^b^	− 0.10

*AUDIT-C* alcohol use disorders identification test-concise, *QoL* quality of life, *WHOQOL-BREF* World Health Organization quality of life-brief version

^a^Correlation is significant at the 0.01 level (two-tailed)

^b^correlation is significant at the 0.05 level (two-tailed)

In males with low risk of alcohol use disorders, alcohol consumption had a moderate positive correlation with psychological QoL. In females with low risk of alcohol use disorders, alcohol consumption was positively but weakly correlated with overall QoL, general health, physical, psychological and environmental QoL domains. There were no significant correlations between alcohol consumption and QoL in males with medium or high risk for alcohol use disorders. In females with a medium risk for alcohol use disorders, there was a positive but weak correlation between alcohol consumption and overall QoL. There were no significant correlations between amount of alcohol consumed and QoL in females with high risk of an alcohol use disorder. In males with severe risk of an alcohol use disorder, there was a negative weak correlation with general health and a negative moderate correlation with social QoL. In females with a severe risk of alcohol use disorders, there were negative but weak correlations between the amount of alcohol consumed and general health, physical, psychological and social QoL domains (Table 3).

### Alcohol consumption level and quality of life by remoteness

AUDIT-C scores were significantly higher in males residing in outer regional, remote and very remote areas than major cities and inner regional Australia (mean difference = -2.4, *p* < 0.01) (Table 4). There was no difference observed between the AUDIT-C scores of females by location. Rurality was not associated with overall QoL, general health, or the domains of physical, psychological and social QoL. Respondents residing in major cities had higher levels of environmental QoL than people residing in outer regional, remote and very remote Australia (mean difference = 6.6, *p* < 0.01). To better understand this result, the responses to individual items from the WHOQOL-BREF Environmental domain were examined and the question with the largest difference between the two locations was question 24: *How satisfied are you with your access to health services?* (Mean difference = 1.09, *p* < 0.01).

**Table 4:** Comparing alcohol consumption and quality of life by rurality in Australia

Variables	Location, mean (SD)			ANOVA	
	Major cities (*n* = 453)	Inner regional (*n* = 1004)	Outer regional, remote and very remote (*n* = 262)	*F*	*p*
AUDIT-C score					
Combined^a^	3.3 (2.8)	3.1 (2.6)	3.4 (3.0)	1.56	0.21
Male	4.1 (3.1)	4.1 (3.0)	6.5 (2.8)	9.10	< 0.01
Female	3.1 (2.7)	3.0 (2.5)	3.0 (2.8)	0.35	0.70
WHOQOL-BREF					
Overall QoL	4.2 (1.0)	4.2 (1.0)	4.2 (0.9)	0.31	0.73
General health	3.4 (1.1)	3.4 (1.2)	3.3 (1.1)	0.51	0.60
Domains					
Physical	65.3 (22.3)	65.0 (22.5)	64.2 (22.7)	0.22	0.81
Psychological	59.8 (20.7)	62.1 (20.1)	60.8 (19.9)	2.12	0.12
Social	61.8 (23.61)	63.8 (23.7)	63.4 (23.2)	1.13	0.32
Environmental	70.9 (18.5)	70.6 (18.8)	64.3 (18.0)	12.86	< 0.01

*AUDIT-C* alcohol use disorders identification test-concise, *WHOQOL-BREF* World Health Organization quality of life-brief version, *QoL* quality of life, *CI* confidence interval QoL

### Quality of life by sociodemographic characteristics and alcohol consumption

Regression analyses identified that household income, education and age were positively related to QoL across all domains. After adjusting for these sociodemographic variables, alcohol consumption was positively related to overall (*B* = 0.04, *p* < 0.01), environmental (*B* = 0.73, *p* < 0.01) and physical QoL (*B* = 0.81, *p* < 0.01); and general health (*B* = 0.04, *p* < 0.01). Remoteness area was only significantly related to environmental QoL (*B* = − 0.25, *p* < 0.01). Females had higher overall QoL and higher social QoL after adjusting for sociodemographic variables (Table 5).

**Table 5:** Multiple linear regression of quality of life by sociodemographic characteristics and alcohol consumption

Independent variable	Overall QoL					Social QoL					Environmental QoL				
	Overall model		R^2^	F	*p*	Overall model		R^2^	F	*p*	Overall model		R^2^	F	*p*
			0.10	27.28	< 0.001			0.05	11.25	< 0.001			0.15	41.53	< 0.001
	*B*	SE	*β*	*t*	*p*	*B*	SE	*β*	*t*	*p*	*B*	SE	*β*	*t*	*p*
Household Income	0.04	0.01	0.21	7.93	< 0.01	0.67	0.14	0.14	4.95	< 0.01	0.78	0.10	0.21	7.85	< 0.01
Education	0.15	0.03	0.15	5.67	< 0.01	2.06	0.67	0.08	3.07	< 0.01	3.64	0.50	0.19	7.35	< 0.01
Age	0.01	0.00	0.05	5.74	< 0.01	0.21	0.04	0.13	4.69	< 0.01	0.36	0.03	0.28	10.97	< 0.01
AUDIT-C score	0.04	0.01	0.13	4.93	< 0.01	0.17	0.23	0.02	0.74	0.46	0.73	0.17	0.19	4.40	< 0.01
Female gender	0.17	0.06	0.06	2.64	< 0.01	7.29	1.63	0.12	4.46	< 0.01	0.40	1.20	0.11	0.33	0.74
Remoteness	− 0.01	0.36	0.01	− 0.38	0.70	0.90	0.93	0.03	0.97	0.33	− 2.52	0.69	− 0.09	− 3.68	< 0.01

Independent variable	General health					Physical QoL					Psychological QoL				
	Overall model		R^2^	F	*p*	Overall model		R^2^	F	*p*	Overall model		R^2^	F	*p*
			0.048	11.99	< 0.001			0.116	31.31	< 0.001			0.098	25.89	< 0.001
	*B*	SE	*β*	*t*	*p*	*B*	SE	*β*	*t*	*p*	*B*	SE	*β*	*t*	*p*
Household Income	0.03	0.01	0.12	4.37	< 0.01	1.06	0.12	0.23	8.56	< 0.01	0.68	0.11	0.16	5.99	< 0.01
Education	0.14	0.03	0.12	4.20	< 0.01	4.10	0.61	0.18	6.69	< 0.01	3.13	0.56	0.15	5.60	< 0.01
Age	0.01	0.00	0.13	4.63	< 0.01	0.08	0.04	0.05	1.92	< 0.05	0.39	0.04	0.28	10.62	< 0.01
AUDIT-C score	0.04	0.01	0.08	3.05	< 0.01	0.78	0.21	0.10	3.79	< 0.01	0.15	0.19	0.02	0.80	0.42
Female gender	− 0.09	0.08	− 0.03	− 1.08	0.28	− 1.09	1.49	− 0.02	− 0.73	0.46	− 1.06	1.36	− 0.02	− 0.79	0.43
Remoteness	0.00	0.05	0.00	0.04	0.97	− 0.08	0.85	0.00	− 0.10	0.92	0.60	0.78	0.02	0.78	0.44

*QoL* quality of life measured through the World Health Organization quality of life-brief version, *AUDIT-C* alcohol use disorders identification test-concise, *B* unstandardised beta, *SE* standard error

## Discussion

This study assessed relationships between alcohol intake and multifaceted QoL in Australia, by gender and risk of alcohol use disorder (low, medium, high, severe). There were significant main effects of AUD risk category on overall QoL, General health and all QoL domains. Physical Health QoL was lower in the low-risk category than all other categories and higher in the moderate than the severe category. Psychological QoL was lower for those in the low than the moderate alcohol risk category and higher in the moderate than the severe category. Environmental QoL was higher in participants in the moderate and severe AUD categories than in the low-risk category. Overall QoL and general health were higher for respondents in the moderate and high than the low AUD categories. General health was lower for those in the severe than the moderate risk AUD category. Overall, these results indicate that those at the lowest and highest (low and severe) levels of alcohol intake had poorer health-related QoL than those with intermediate consumption, classified at medium–high risk of alcohol use disorder in the AUDIT-C.

Overall, the correlations between alcohol consumption and QoL were positive in those at low risk of an alcohol use disorder, non-significant or positive for those at medium or high risk of an alcohol use disorder, and negative for those at severe risk of alcohol use disorder. Mean QoL scores were somewhat higher for moderate-to-high risk drinkers than low and severe risk drinkers. This finding may be related to the Australian drinking culture, as the consumption of alcohol is integral to many social activities [39]. Several studies conducted in other settings found an inverted U-shaped relationship between alcohol use and QoL, with higher QoL for those with moderate drinking [3, 8, 40]. By contrast, a study conducted in India which used the same scales as this study (AUDIT and WHOQOL-BREF questionnaires) found a linear relationship where QoL was significantly lower across all subscales for alcohol drinkers compared with non-drinkers [4]. However, this was an all-male study with a smaller sample size (*n* = 316). Therefore, the current study found somewhat higher self-reported QoL in those with moderate compared to the lowest or highest alcohol consumption in the Australian context.

Male participants had higher AUDIT-C scores than females. Approximately 20% of males and 8% of females in this study were in the severe risk range for alcohol use disorders. This was consistent with the latest National Health Survey by the Australian Bureau of Statistics which reported that a higher percentage of males than females exceeded the lifetime risk guidelines for alcohol intake [16]. Therefore, as males have a higher risk for alcohol use disorders, it is important to determine the contributing factors for such consumption levels and target these behaviours in health strategies to reduce negative outcomes.

Male participants residing in outer regional, remote or very remote Australia (RA3, RA4 and RA5) had significantly higher alcohol intake than males in major cities and inner regional areas (RA1 and RA2). This is consistent with previous research and could be due to perceived social benefits, such as feeling included and participation in community events [22]. Regional and rural areas tend to have reduced access to alcohol treatment options and fewer medical professionals resulting in fewer opportunities for advice on harm-minimisation [25]. The current study found that participants residing in outer regional, remote and very remote Australia reported lower satisfaction with access to health services than respondents residing in major cities, which supports a possible link between fewer opportunities for treatment and advice and higher alcohol intake. The Australian Institute of Health and Welfare (2018) reported that reduced access to medical services and increased disease risk factors has resulted in an increase in total disease burden per population (expressed as disability-adjusted life years) and a higher rate of potentially avoidable deaths and hospitalisations in people residing in remote and very remote communities in Australia compared to people residing in major cities [41].

Regression analyses identified that household income, education and age were positively related to QoL across all domains. After adjusting for these sociodemographic variables, alcohol consumption was positively related to overall QoL, environmental and physical QoL and general health. Overall and social QoL were higher in males than females after adjustment for sociodemographic variables. While alcohol had an overall positive relationship with QoL, it is worth noting that several studies have shown that alcohol, even for those at low risk of harm, can have negative health effects, including higher risk of trauma, hypertension, dementia, some cancers and a range of other health conditions [1, 2, 42, 43]. Additionally, it should be borne in mind that relationships between household income and education were consistently stronger predictors of QoL than alcohol intake.

This study has several limitations. Firstly, the study design is cross sectional, and therefore, the directional relationship between alcohol use and QoL cannot be determined. Furthermore, this study only considered alcohol use, QoL, gender, income, education and rurality. Other contributing factors such as medical or psychiatric morbidity or cultural factors in alcohol use were not assessed. Those with serious or life-threatening medical conditions, who are likely to have lower QoL, may abstain from alcohol; in one study, abstainers had higher mortality than drinkers, with many of them being previously high-risk drinkers [44]. Similarly, personality traits were not examined; for instance, it is possible that individuals with higher-rated QoL may also be more gregarious or community-minded and therefore use alcohol within a social context. Those with high risk of alcohol use disorders may experience physiological dependence, which may influence alcohol intake. The study was part of a larger health survey and drinking motives were not assessed. We note that there is a lack of research of alcohol intake and QoL that assesses drinking motives. Further, qualitative studies may be useful to better understand the differences in alcohol consumption.

Additionally, the study was online which could have resulted in responder bias by including only participants who utilise social media [45]. The current sample had a smaller proportion of people living in major cities and a larger proportion of those in regional areas than the Australian population overall [46] and an overrepresentation of female participants (83.1%). Previous research indicates that females are more likely to volunteer for surveys both through social media and through the other modes of recruitment [45]. Therefore, further studies that seek to recruit a nationally representative sample are desirable. However, the current study to our knowledge has a more extensive sample than previous studies of alcohol consumption and health-related QoL in the Australian context.

The study overlapped with the COVID-19 pandemic, which likely affected both QoL and alcohol intake. It may also have affected enrolment in the study. Preliminary research examining the impact of the pandemic on alcohol intakes has been mixed, showing both reductions and increases in different countries and groups [47]. Further research is needed to understand the generalisability of the findings and longer-term trends.

In summary, we found that relationships between alcohol consumption and health-related QoL are multifaceted and differ by alcohol risk category, gender and rurality. Alcohol consumption in the severe risk range correlates with lower QoL across several domains. After adjusting for sociodemographic variables, alcohol consumption is positively related to overall, environmental and physical QoL and general health. Males consume more alcohol than females and are more likely to drink at riskier levels. Furthermore, males in outer regional, remote and very remote areas consume more alcohol than males in major cities and inner regional areas. The current results are consistent with and extend upon previous studies showing the importance of gender and rurality in relation to alcohol consumption in Australia.

## Conclusions

The results provide further information about relationships between alcohol intake and health in Australia that can be used to help inform prevention, screening and delivery of interventions. Future research should explore factors contributing to higher-risk alcohol consumption in males, particularly those residing in regional, rural and remote Australia. The current results support the need for further research and health resources for those residing in regional, rural and remote Australia.

## Data Availability

Data are available on reasonable request from the corresponding author.
